# The Hormone Therapy and Constipation

**Published:** 1914-06

**Authors:** 


### ABSTRACTS.

---

**The Hormone Therapy and Constipation.** Juan Raul Goyena, La Semana Medica, July 17, 1913, discusses the hormone theory and gives the histories of thirteen cases of constipation in which he has used Horomal. The word hormone is taken from the Greek (I excite) and as Bayliss and Starling have pointed out, the substances formed in the glands are taken up by the circulation and carried to certain organs, the functions of which they excite. In 1904 Enriquez and Hallion observed that if an extract of the duodenal mucosa is injected into an animal, the pancreatic secretion is excited and a stimulation of the peristaltic contractions results. In 1908 Zuelger, Dohm and Marxer discovered this substance in the stomach also. Later, they found the substance in the spleen which simplified greatly the work of procuring a sterile preparation. Horomal, is apparently carried to the spleen by the circulation and there stored.

It is prepared in sterile capsules of 20 C.C. each—this being the average dose for intravenous or intramuscular injection. In the latter case, Beta-eucaine is added to prevent pain. The indications for its administration—according to the author—

are clear. "In all cases in which the contractibility of the intestine is found paralyzed or abolished, it will be useful." It is most useful in acute paralysis of the bowel and in chronic constipation. To assist in cleaning the bowel of the impacted feces, he gives castor oil simultaneously with the first injection. The injections will probably have to be repeated, although it is claimed that a single injection will accomplish the desired result and that its action will persist for a considerable time.

